# Esophageal Diameter as a Function of Weight in Neonates, Children and Adolescents: Reference Values for Dilatation of Esophageal Stenoses

**DOI:** 10.3389/fped.2022.822271

**Published:** 2022-02-28

**Authors:** Steffan Loff, Oliver Diez, Wei Ho, Thekla v. Kalle, Svetlana Hetjens, Michael Boettcher

**Affiliations:** ^1^Kinderchirurgische Klinik, Olgahospital, Klinikum Stuttgart, Stuttgart, Germany; ^2^Radiologisches Institut, Olgahospital, Klinikum Stuttgart, Stuttgart, Germany; ^3^Medizinische Statistik, Biomathematik und Informationsverarbeitung Universitätsmedizin Mannheim, Mannheim, Germany; ^4^Department of Pediatric Surgery, University Medical Center Mannheim, Heidelberg University, Mannheim, Germany

**Keywords:** esophageal stenosis, esophageal atresia, burn of esophagus, dilatation, diameter of normal esophagus

## Abstract

**Introduction:**

Esophageal stenoses are frequent complications after esophageal atresia surgery as well as after acid, alkali and battery ingestion. Worldwide, repeated balloon dilatations are the most frequently performed procedures for these stenoses. In most cases the stenoses can be dilated sufficiently to allow adequate enteral nutrition. Until recently, age dependent esophageal lumen size has not been established; which was aim of the current study.

**Methods:**

All children in whom an esophageal contrast imaging was performed between 1/2011 and 5/2021 were included. The width was measured by two investigators at two measuring points in two planes, the diameter was calculated and plotted against the respective weight of the child. Bland-Altmann plots have confirmed the validity of the measurements of both investigators.

**Results:**

Esophagus diameter was measured in more than 100 children. The resulting curves show a very good correlation with weight (upper measuring point: *r* = 0.86743, *p* < 0.0001; lower measuring point: *r* = 0.80593, *p* < 0.0001).

**Conclusion:**

These results are the first to define the esophageal diameter in children. The results of this study may guide physicians performing esophageal interventions including dilatations in future.

## Introduction

Esophageal stenoses in childhood usually result from accidental acid or alkali burns, battery ingestions, or as a result of esophageal reconstruction in children with esophageal atresia.

Acid burns usually occur when children accidentally ingest acidic or alkalic fluids. The severity of the expected stenoses varies from nonexistent to severe narrowing (1). Treatment of severe stenoses often consists of repeated balloon dilatations or bougienage (2, 3) until the esophagus has stabilized at an adequate lumen size. If this cannot be achieved with dilatations and additional measures such as cortisone injections, mitomycin overlays or scar incisions, the stenosis must be resected and reanastomosed and in some cases an esophageal replacement using a gastric or intestinal interposition is necessary to avoid multiple dilatation procedures, which produce their own morbidity (4–6).

When infants swallow button cell batteries they need to be removed endoscopically very quickly to avoid severe local burns, often transmural. In such cases, a locally circumscribed stenosis of the esophagus may occur (7, 8). These stenoses are also usually treated as described above.

After surgical correction for esophageal atresia, narrowing of the anastomosis occurs in 30–70% of cases (9–12). Again, the primary therapy is usually balloon dilatation, with cortisone injections or mitomycin-C overlays if necessary (13, 14). However, bougienage and stent implantation as well as stenosis resection and esophageal replacement procedures are also used (15–17).

For that reason, dilatation of esophageal strictures by balloon dilatation or bougienage is a very common procedure. While the basic procedure of dilatation treatment is always similar, details vary. One can treat only symptomatic stenoses or all those that are not of normal width (18). One can perform the procedure endoscopically controlled, radiologically controlled, or both. Moreover, the dilatation steps for a narrow stenosis are managed differently. And there are no reference values for the target size of the esophageal lumen.

A typical but rare complication of esophageal stricture dilatation is perforation of the esophagus. It occurs with a frequency of 0.4–17% (2, 17, 19, 20). For the most part, perforations heal spontaneously. However, the course may be complicated by severe mediastinitis. Deaths may also occur (21).

Surprisingly, no age or weight-dependent reference values for esophageal width have been established, guiding dilatation therapies in children. In order to determine natural esophagus width, one may use contrast radiographs of the esophagus with visualization of the lumen for the entire length. We recently published a first study of esophageal width using this technique. However, due to the small sample size, generability was limited especially for older children (22). Thus, the aim of the current study was to determine esophageal width in a large cohort of children and to establish reference values for children.

## Materials and Methods

In a retrospective study, all radiographic esophageal contrast-imaging of newborns, children and adolescents up to 18 years of age between 1/2011 and 5/2021 were studied.

Exclusion criteria were:

- Patients below the 3rd and above the 97th weight percentiles.
- Patients who have had disease of the esophagus itself, such as esophageal atresia or achalasia.
- Examinations that generally did not show good contrast of the esophagus.
- Examinations in which the upper and lower measuring points were not equally assessable.
- Examinations in which the esophagus was not well visualized in both planes.
- Examinations in which the act of swallowing with fluoroscopic visualization of the propulsion was not shown were also excluded, since the maximum width of the esophagus can only be assessed when a propulsion wave is passed.
- Examinations in which no mm scaling was applied.

Limited by these strict exclusion criteria, 108 patients could nevertheless be included in the study. These patients had contrast examinations mainly to exclude gastro-esophageal reflux or foreign body ingestion. All were judged as normal esophagograms.

**Figure 1:**
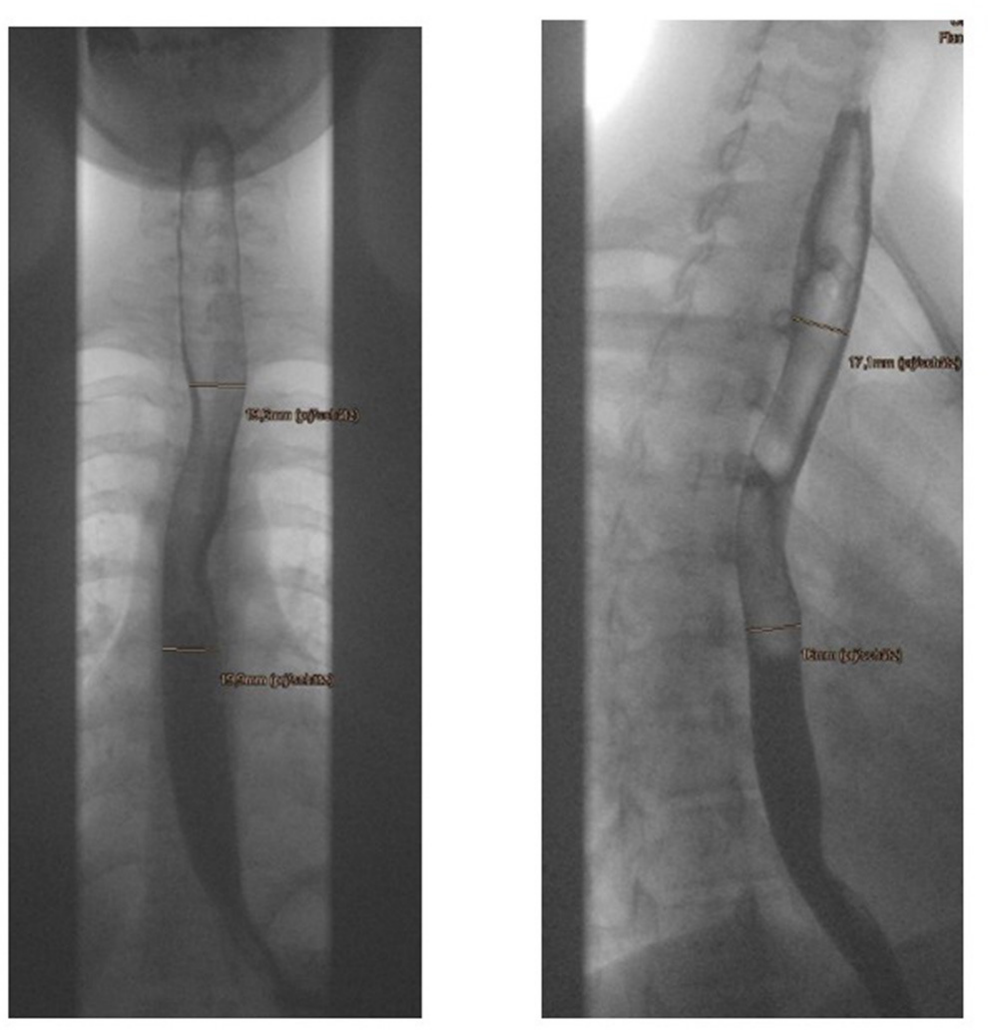
Upper and lower measuring points in both planes.

The width of the esophagus was measured at two levels: The projection of the upper edge of the 3rd thoracic vertebra was determined as the upper measuring point, and the projection of the upper edge of the 7th thoracic vertebra was determined as the lower measuring point. The reason for the two measuring points was that the esophagus is generally somewhat narrower in the upper region than in the lower region and we wanted a statement regarding the entire esophagus. The upper measuring point, on the other hand, is particularly relevant for children with stenoses after esophageal atresia, since in most cases the anastomosis is located at this level. At both measuring points, the width of the esophageal lumen was measured in two planes. The width was determined without exception on the basis of video-fluoroscopic motion studies, so that it was possible to follow the actual bolus and determine the maximum width at the mentioned measuring points (Figure 1).

All four measurements for each patient were taken by two blinded examiners. The mean values were formed from the measurements of the two examiners, so that four averaged values were then available per patient, in each case a.p. and laterally at the upper and lower measuring point. Then, the mean values were calculated from the a.p. and lateral measurements at both sites to obtain the mean diameter at each site. The values obtained were statistically evaluated and formed the basis for the curves and the tables.

All statistical calculations were performed using SAS software, release 9.4 (SAS Institute Inc., Cary, NC, USA). Correlation values for upper and lower measuring points with weight were determined by calculating Pearson's correlation coefficient. The regression analysis was performed to predict the mean esophageal diameter at each measuring point. A *p*-value of <0.05 was considered statistically significant.

## Results

**Figure 2:**
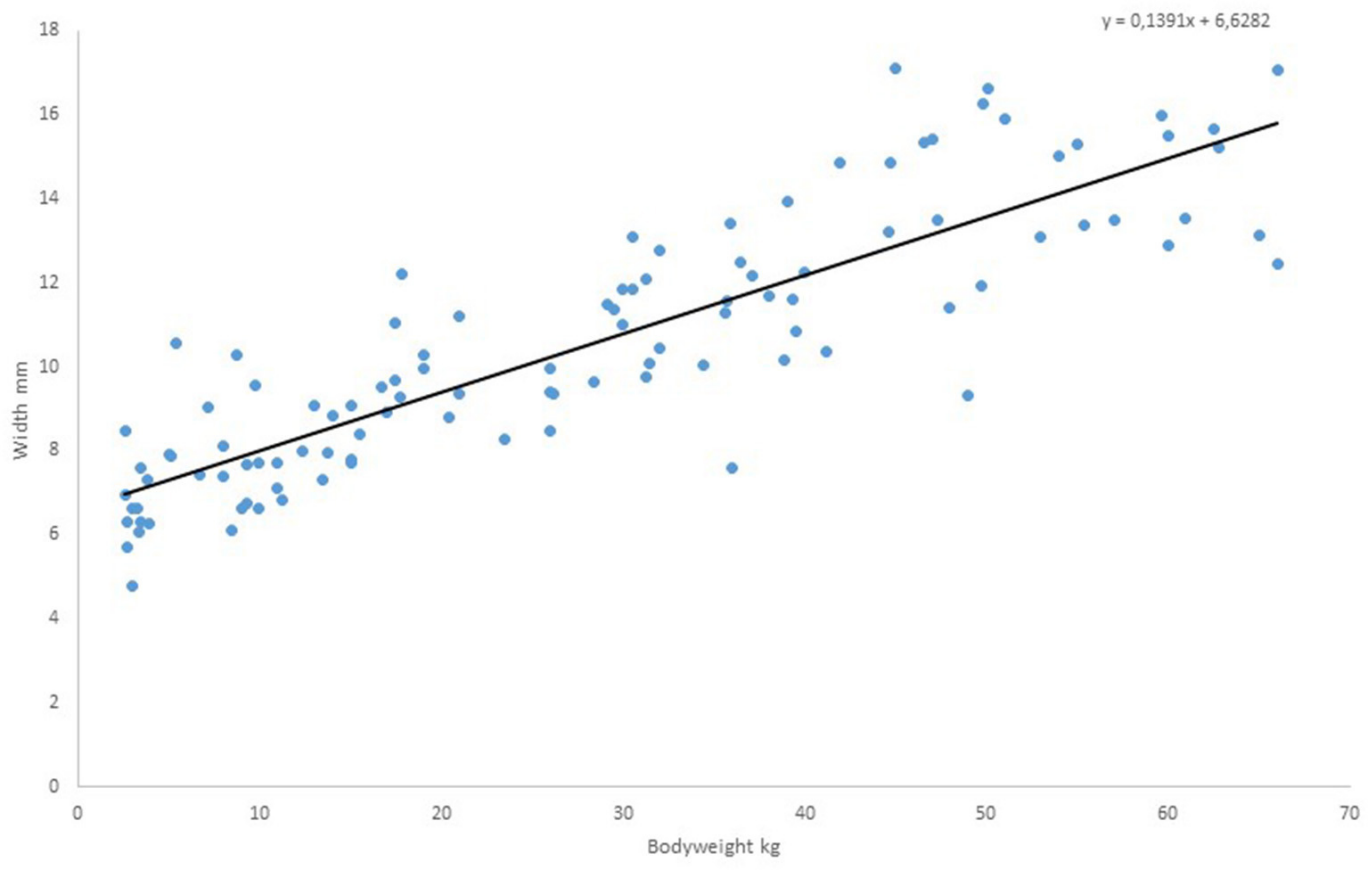
Measured values of diameter of the esophagus as a function of body weight, upper measuring point.

Figure 2 shows the measured diameter of the esophagus as a function of body weight at the upper measuring point at the upper edge of the 3rd thoracic vertebra. For the regression line *y* = 0.1391x + 6.6282 there is a high correlation of *r* = 0.86743, *p* < 0.0001. Nevertheless, significant deviations from the mean value of esophageal diameter at the corresponding body weights are evident. Thus, the diameter of the esophagus in the 3-kg children varies between 4.8 and 8.48 mm. There is a range of variation of 1.5–2 mm up and down from the regression line over the entire graph, except for a few outliers. There are clearly downward outliers at the three weight points 36, 49, and 66 kg. It may be assumed that in these cases the esophagus was not filled asto the maximum.

**Figure 3:**
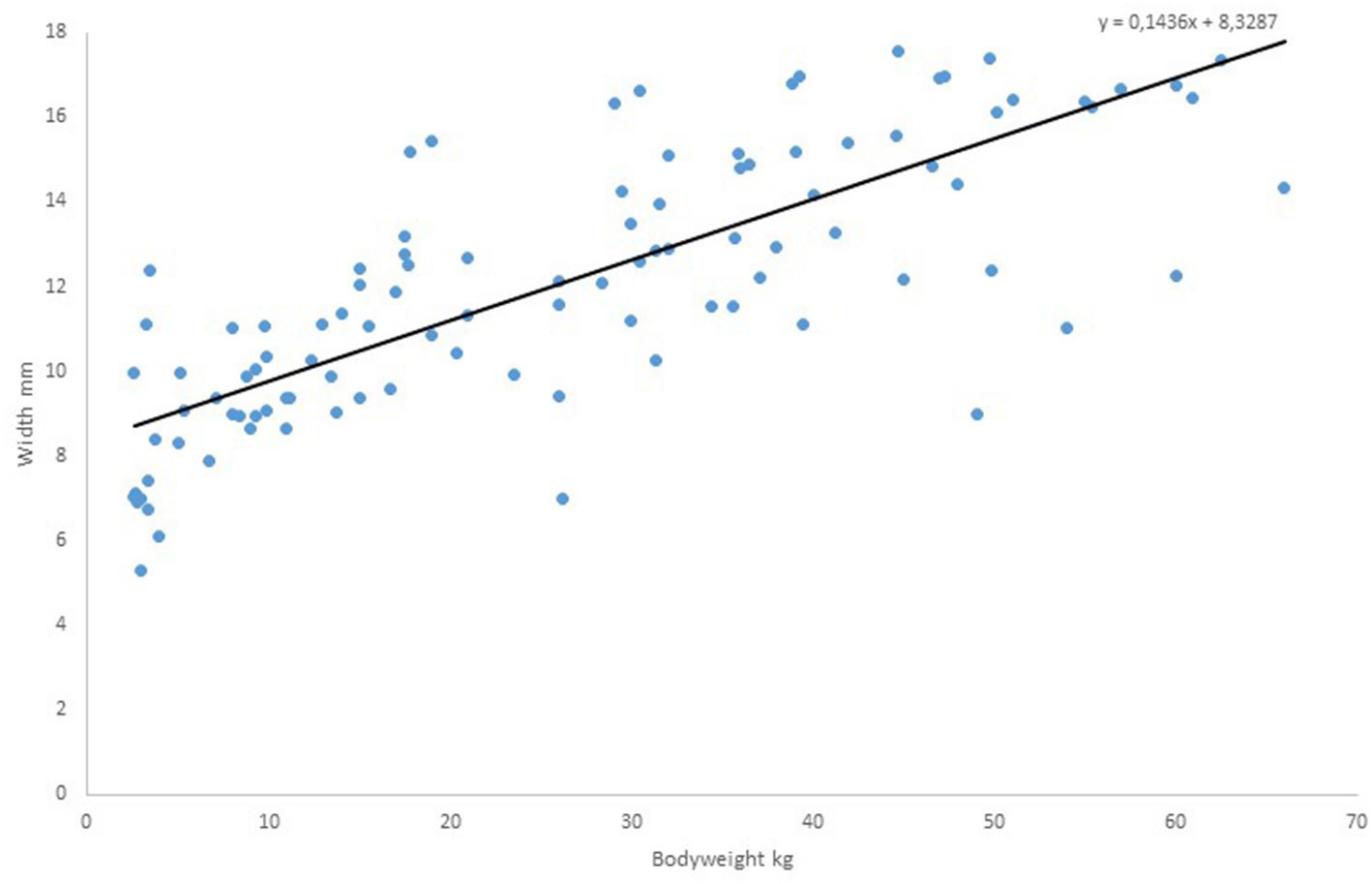
Measured values of diameter of the esophagus as a function of body weight, lower measuring point.

Figure 3 shows the measured diameter of the esophagus as a function of body weight at the lower measuring point, the upper edge of the 7th thoracic vertebra. Again, we find a high correlation with the regression line *y* = 0.1436x + 8.3287 of *r* = 0.80593, *p* < 0.0001. Obvious outliers are somewhat more common in the 2nd graph. Apart from these, the variance of esophageal diameter around the regression line is also 1.5–2 mm.

From the linear equation of the regression line, the esophageal diameter is derived as a function of weight in kg. This was done in Table 1 for the upper measuring point. The table is detailed to allow the operator to find the correct diameter at a glance. This measuring point has a special significance for the dilatation of esophageal stenosis after esophageal atresia.

**Table 1:** Mean diameter of the esophagus as calculated by regression line, upper measuring point.

**kg**	**mm**	**kg**	**mm**	**kg**	**mm**	**kg**	**mm**
3	7	21	9.5	41	12.3	61	15.1
4	7.2	22	9.7	42	12.5	62	15.3
5	7.3	23	9.8	43	12.6	63	15.4
6	7.5	24	10	44	12.7	64	15.5
7	7.6	25	10.1	45	12.9	65	15.7
8	7.7	26	10.2	46	13	66	15.8
9	7.9	27	10.4	47	13.2	67	15.9
10	8	28	10.5	48	13.3	68	16
11	8.2	29	10.7	49	13.4	69	16.2
12	8.3	30	10.8	50	13.6	70	16.4
13	8.4	31	10.9	51	13.7		
14	8.6	32	11.1	52	13.9		
15	8.7	33	11.2	53	14		
16	8.9	34	11.4	54	14.1		
17	9	35	11.5	55	14.3		
18	9.1	36	11.6	56	14.4		
19	9.3	37	11.8	57	14.6		
20	9.4	38	11.9	58	14.7		
		39	12.1	59	14.8		
		40	12.2	60	15		

In Table 2, the same calculation is found for the lower measuring point at the level of the upper edge of the 7th thoracic vertebra. The values are somewhat higher, indicating that the esophagus in this area generally has a somewhat wider lumen than further up.

**Table 2:** Mean diameter of the esophagus as calculated by regression line, lower measuring point.

**kg**	**mm**	**kg**	**mm**		**kg**	**mm**	**kg**	**mm**
3	8.8	21	11.3		41	14.2	61	17.1
4	8.9	22	11.5		42	14.4	62	17.2
5	9.1	23	11.6		43	14.5	63	17.4
6	9.2	24	11.8		44	14.7	64	17.5
7	9.3	25	11.9		45	14.8	65	17.7
8	9.5	26	12.1		46	14.9	66	17.8
9	9.6	27	12.2		47	15.1	67	18
10	9.8	28	12.4		48	15.2	68	18.1
11	9.9	29	12.5		49	15.4	69	18.2
12	10.1	30	12.6		50	15.5	70	18.4
13	10.2	31	12.8		51	15.7		
14	10.3	32	12.9		52	15.8		
15	10.5	33	13.1		53	15.9		
16	10.6	34	13.2		54	16.1		
17	10.8	35	13.4		55	16.2		
18	10.9	36	13.5		56	16.4		
19	11.1	37	13.6		57	16.5		
20	11.2	38	13.8		58	16.7		
		39	13.9		59	16.8		
		40	14.1		60	17		

## Discussion

Esophageal stenosis is a common problem for those dealing with esophageal atresia and esophageal burns. Various esophageal target diameter have been reported. Balloon diameters between 4 and 20 mm are given depending on the surgeon's assessment (3, 15, 23). Often there is no information available or it is vague, such as the “rule of the thumb” (6). Other investigators specify a maximum diameter of 10 mm (24) or 14 mm (17). Excessive dilatation of the esophagus beyond the natural lumen implies an inherent risk of perforation. Wei-Zhong's group reports a rather high rate of perforation during balloon dilatations, which they also explain by the fact that a too high target diameter above 10 mm was used. They therefore suggest the use of balloon catheters with a lumen below 10 mm in children under 2 years of age (20). From these publications, there is a clear mandate to determine the natural diameter of an esophagus at different body weights. This would be the only way to avoid gross under- or over-dilatation at a given body weight. However, systematic measurement of normal esophageal lumina has not been performed. In the current study weight-dependent esophageal reference values have been defined which could guide future dilatation procedures in children. The mean diameter of the esophagus, indicated by the regression lines or the tables, may give a good approximation of the target diameter to aim for. Radiologists, gastroenterologists, and pediatric surgeons who treat esophageal stenoses with dilation, bougienage, or stenting may use it as a guide to avoid gross overdilatation with the risk of perforation.

## Limitations

Due to the variability of the measurements, this correlation of weight to esophageal diameter should be regarded as landmark values with a tolerable variation ±1.5–2 mm rather than absolutes. The relatively broad spread must be interpreted as the natural fluctuation of the esophageal lumen size at a given weight. Moreover, these esophageal diameter measurements rely on the swallowing of fluid contrast medium which may underestimate esophageal lumen. It is true that only studies that had fluoroscopically imaged a bolus passage were used and thus a maximum diameter was measured under these conditions. Nevertheless, we do not know whether the esophagus would dilate further with the use of pasty contrast medium.

In general, it should of course be noted that the expansion should be carried out in small steps in order to avoid rupture. At our clinic, therefore, we generally do not dilate more than 2 mm beyond the current diameter in a single session. This has been reported previously (6, 20, 25).

## Conclusion

The results of this study are the first to define the normal esophageal diameter and it's variation in children. They may guide physicians performing esophageal interventions including dilatations in future.

## Data Availability Statement

The original contributions presented in the study are included in the article/supplementary material, further inquiries can be directed to the corresponding author.

## Ethics Statement

Ethical review and approval was not required for the study on human participants in accordance with the local legislation and institutional requirements. Written informed consent from the participants' legal guardian/next of kin was not required to participate in this study in accordance with the national legislation and the institutional requirements.

## Author Contributions

SL: investigator of x-ray-examinations and writing of text. OD: investigator of x-ray-examinations and correction of text. WH: recruitment of data. SH: statistcs. TK: providing of x-ray-examinations and text writing. MB: text writing and corrections.

## Conflict of Interest

The authors declare that the research was conducted in the absence of any commercial or financial relationships that could be construed as a potential conflict of interest.

## Publisher's Note

All claims expressed in this article are solely those of the authors and do not necessarily represent those of their affiliated organizations, or those of the publisher, the editors and the reviewers. Any product that may be evaluated in this article, or claim that may be made by its manufacturer, is not guaranteed or endorsed by the publisher.
